# Inflammation promotes stomach epithelial defense by stimulating the secretion of antimicrobial peptides in the mucus

**DOI:** 10.1080/19490976.2024.2390680

**Published:** 2024-09-08

**Authors:** Megi Vllahu, Antonia Voli, Valerio Licursi, Claudia Zagami, Antonella D’Amore, Jan Traulsen, Sara Woelffling, Monika Schmid, Robbie Crickley, Richard Lisle, Alexander Link, Alessandra Tosco, Thomas F. Meyer, Francesco Boccellato

**Affiliations:** a Department of Pharmacy, University of Salerno, Fisciano, Salerno, Italy; b Department of Molecular Biology, Max Planck Institute for Infection Biology, Berlin, Germany; c Ludwig Institute for Cancer Research, Nuffield Department of Clinical Medicine, University of Oxford, Oxford, UK; d Institute of Molecular Biology and Pathology (IBPM), National Research Council (CNR) of Italy c/o Department of Biology and Biotechnology ‘‘C. Darwin’’, Sapienza University, Rome, Italy; e Department of Gastroenterology, Hepatology and Infectious Diseases, Otto-von-Guericke University Hospital, Magdeburg, Germany; f Laboratory of Infection Oncology, Institute of Clinical Molecular Biology, Christian Albrecht University of Kiel and University Hospital Schleswig-Holstein - Campus Kiel, Kiel, Germany

**Keywords:** Mucus, Inflammation, Helicobacter pylori, Antimicrobial peptides, Stomach, Innate immunity, Epithelial defence

## Abstract

The mucus serves as a protective barrier in the gastrointestinal tract against microbial attacks. While its role extends beyond merely being a physical barrier, the extent of its active bactericidal properties remains unclear, and the mechanisms regulating these properties are not yet understood. We propose that inflammation induces epithelial cells to secrete antimicrobial peptides, transforming mucus into an active bactericidal agent. To investigate the properties of mucus, we previously developed mucosoid culture models that mimic the healthy human stomach epithelium. Similar to organoids, mucosoids are stem cell-driven cultures; however, the cells are cultivated on transwells at air–liquid interface. The epithelial cells of mucosoids form a polarized monolayer, allowing differentiation into all stomach lineages, including mucus-secreting cells. This setup facilitates the secretion and accumulation of mucus on the apical side of the mucosoids, enabling analysis of its bactericidal effects and protein composition, including antimicrobial peptides. Our findings show that TNFα, IL1β, and IFNγ induce the secretion of antimicrobials such as lactotransferrin, lipocalin2, complement component 3, and CXCL9 into the mucus. This antimicrobial-enriched mucus can partially eliminate *Helicobacter pylori*, a key stomach pathogen. The bactericidal activity depends on the concentration of each antimicrobial and their gene expression is higher in patients with inflammation and *H.pylori*-associated chronic gastritis. However, we also find that *H. pylori* infection can reduce the expression of antimicrobial encoding genes promoted by inflammation. These findings suggest that controlling antimicrobial secretion in the mucus is a critical component of epithelial immunity. However, pathogens like *H. pylori* can overcome these defenses and survive in the mucosa.

## GRAPHICAL ABSTRACT

**Figure:**
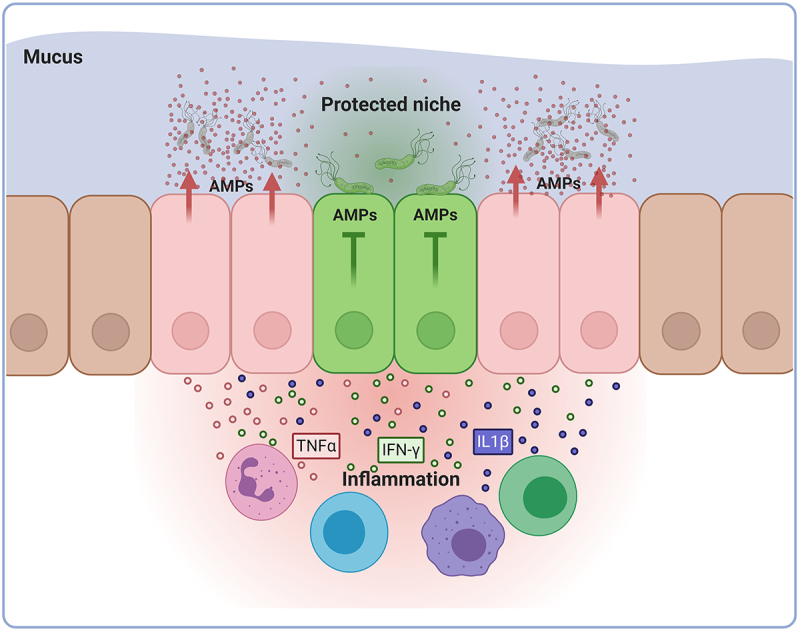

## Introduction

The gastrointestinal epithelium is a dynamic barrier that separates the host from potentially harmful microbes ingested with food. This barrier is maintained through a balance of physical structures (glands, crypts), immune cells, and secreted factors, including mucus and antimicrobial-peptides (AMPs).^1,2^ Mucus is a complex mixture of glycoproteins and other molecules which provides a physical barrier^3^ and a unique environment for the interaction with microorganisms.^4^ Experiments in animal models suggest that microbial control in the gut takes place in the mucus, as antimicrobial activity is restricted to the mucosal environment and does not extend to the gut lumen.^5^ AMPs are small proteins that play a crucial role in the innate immune response across various organisms, including mammals, insects, plants, and bacteria.^6,7^ These polypeptides are known for their broad-spectrum antimicrobial activity against bacteria, fungi, viruses, and even some parasites.^8^ AMPs are usually amphipathic: their hydrophobic residues mediate insertion into bacterial membranes, while clusters of positive residues facilitate membrane disruption and bacterial death.^9^ Within the mucosa, AMPs such as defensins and cathelicidins play a vital role in controlling microbial populations and maintaining homeostasis.^10^ Their expression usually correlates to infections or inflammation of the mucosa.

*Helicobacter pylori* is a Gram-negative bacterium with a unique tropism for the human stomach, where it is the only known colonizer.^11^ Infection is associated with various gastrointestinal diseases, including ulcers and gastric cancer.^12^ This unique host–pathogen interaction provides an opportunity to understand the epithelial host response to infection, set against the backdrop of the complex interactions of microbial communities that are typically found in the lower part of the gut. In infected human stomachs it has been reported the expression of AMPs such as human-beta-defensins^13,14^ and Cathelicidins such as LL-37^15^, while in infected mouse models it has been reported the expression of intellectin-1.^16^

However, there is little direct evidence of how the epithelium regulates secretion of AMPs into the mucus. Moreover, the overall efficacy of mucus in defending against specific pathogens has not been measured. This limitation is mainly due to the lack of *in vitro* models of cells producing native mucus to directly investigate the secreted AMPs and their bactericidal capacity. We have previously developed a stem cell driven model of the healthy human stomach called “mucosoid cultures”.^17^ Similarly to organoids, the epithelial cells of the mucosoids can regenerate indefinitely by preserving their stem cell niches.^17^ Using different cultivation cocktails, these unique cultures display features of the human gastric epithelium *in-situ* including secretion of pepsin, gastric acid, and hormones.^17,18^ Importantly, cells in these cultures can be induced to differentiate into mucus-producing cells and accumulate mucus on the apical side.^17,18^ This unique feature makes mucosoids an excellent model in which to study the impact of this poorly accessible body fluid in controlling microbial invasion. Herein, we take advantage of the unique capabilities of mucosoid cultures to investigate the secretion of AMPs into the mucus during inflammation, and the interplay of this with *H. pylori*.

## Material and methods

### Human stomach

The epithelial cells used in this study to generate mucosoids are derived from weight loss surgery conducted at the Centre for Obesity and Metabolic Medicine, Helios Klinikum, Berlin-Buch, Germany (Table 1). These procedures were carried out with the approval of the ethics committee of Charité University Hospital, Berlin (EA1/129/12). Prior to their inclusion in the study, each participant provided written informed consent. Anonymized tissue samples were collected from patients, with a body mass index (BMI) greater than 36 kg/m.^2^ To prepare the samples, the tissue was first rinsed in phosphate-buffered saline (PBS) containing 50 μg/mL gentamicin in order to remove any blood. Subsequently, excess fat and connective tissue were carefully removed with a scalpel. From each sample, a 2 × 2 cm^2^ section was excised from the central part of the antrum for further analysis.

**Table 1.** Weight loss surgery list of patients.

Code	Date of isolation	Age	Gender	Comments
GAT17	2015-07-14	30	Female	BMI: 53, *H.pylori* unknown status
GAT23	2016-04-15	55	Female	BMI:45, *H.pylori* negative
GAT27	2017-05-10	36	Female	BMI:69, *H.pylori* negative, Diabetes II
GAT28	2017-05-16	32	Male	BMI: 43, *H.pylori* negative
GAT29	2017-05-16	43	Female	BMI: 48, *H.pylori* negative
GAT31	2017-04-12	53	Female	BMI:58, *H.pylori* negative

The gastric biopsies used in this study (Figure 4) were collected from patients undergoing upper gastrointestinal endoscopy at the Department of Gastroenterology, Hepatology and Infectious Diseases at the Otto-von-Guericke University Magdeburg, Germany (Table 2). The study protocol was approved by the Institutional Review Board of Otto-von-Guericke University Magdeburg (ethics reference 80/11). The detailed study design was described previously.^19^ Briefly, every participant provided written informed consent before study inclusion. A pair of biopsies was immediately snap-frozen in liquid nitrogen and stored at −80°C until further processing and extraction of nucleic acids. Additional biopsies from the gastric antrum, the incisura angularis, and the gastric corpus were processed for routine histopathology assessment according to the updated Sydney classification.^20^ This includes grading of the activity of the mucosal inflammation by assessment of the infiltration by neutrophil granulocytes and the chronicity of inflammation by assessment of the infiltration by lymphocytes accordingly. According to the updated Sydney system, *Helicobacter pylori* density was assessed by histopathology. Status of *H. pylori* infection was determined at the clinic by rapid urease test and microbiology (culture), as well as serology (anti *H.pylori* IgM and IgG titer). Patients were defined as *H*. *pylori–*positive with positive microbiology and/or positive histology and/or positive serology. Positive serology only indicates past rather than active infection. Patients negative in these modalities were defined as *H*. *pylori–*negative. In case of inflammation but no structural changes of the mucosa, the sample was classified as Chronic gastritis (CG). In case of no inflammation, no structural changes and no *H.pylori* infection, the sample was classified as normal (N).

**Table 2.** Clinical biopsies data.

						Pathology Biopsy				Grading	
ID	Sex	Age [yrs]	Group	*H. pylori* status	Side of the stomach	Activity	Chronicity	Atrophy	IM	OLGA	OLGIM
33	m	20	*N*	0	Corpus	0	0	0	0	0	0
40	f	65	*N*	0	Corpus	0	1	0	0	0	0
48	f	88	*N*	0	Corpus	0	0	0	0	0	0
55	m	61	*N*	0	Corpus	0	0	0	0	0	0
56	f	31	*N*	0	Corpus	0	0	0	0	0	0
68	m	52	*N*	0	Corpus	0	0	0	0	0	0
56	f	31	*N*	0	Antrum	0	1	0	0	0	0
60	f	40	*N*	0	Antrum	0	1	0	0	0	0
27	f	60	CG	2	Corpus	0	2	0	0	0	0
61	f	51	CG	2	Corpus	1	2	0	0	0	II
98	f	19	CG	2	Corpus	1	1	0	0	0	0
99	m	42	CG	2	Corpus	2	2	0	0	0	0
111	f	46	CG	2	Corpus	2	2	0	0	0	0
121	f	51	CG	2	Corpus	2	2	0	0	0	I
122	f	56	CG	2	Corpus	1	1	0	0	0	0
27	f	60	CG	2	Antrum	0	2	0	0	0	0
99	m	42	CG	2	Antrum	1	2	0	0	0	0
108	f	35	CG	2	Antrum	2	2	0	0	0	0
110	f	47	CG	2	Antrum	2	2	0	0	0	0
122	f	56	CG	2	Antrum	1	2	0	0	0	0
126	f	70	CG	2	Antrum	1	1	0	0	0	0
128	f	33	CG	2	Antrum	1	2	0	0	0	0
129	m	54	CG	2	Antrum	2	2	0	0	0	0
141	m	47	CG	2	Antrum	0	2	0	0	0	0

Abbreviations and coding: f = female, *m* = male; Group: AG = atrophic gastritis, CG = chronic gastritis without atrophy, *N* = normal without inflammation; *H.pylori* status: 0 = negative, 1 = serology positive, 2 = serology and direct proof by either histology, microbiology or rapid urease test. See main text for scoring of the other parameters as well as OLGA/OLGIM staging.

### Generation and maintenance of the mucosoid cultures

Gastric tissue from sleeve resections was processed as described previously. The tissue (0.5*–*1 cm^2^) from antrum and from corpus was cut into pieces <1 mm^2^ and washed in cold PBS until the supernatant was clear (8–10 times), followed by a 30-min incubation in chelating solution (5.6 mM Na_2_HPO_4_; 8.0 mM KH_2_PO_4_; 96.2 mM NaCl; 1.6 mM KCl; 43.4 mM sucrose; 54.9 mM d-sorbitol; 0.5 mM dl-dithiothreitol; 2 mM EDTA in H_2_O) at 37°C on a shaking platform. Settled tissue fragments were transferred to a Petri dish and subjected to gentle pressure under a glass slide to extract the glands. The extracted portions of tissue were re-suspended in medium containing 10% heat-inactivated fetal calf serum (Biochrom) to reduce cell re-aggregation. After settling the larger fragment for 1 min, the solution with the isolated glands was transferred into a new tube, centrifuged at 250x*g* for 5 min and re-suspended in 600 μL of Matrigel (356231; Corning), and aliquoted in 50 μL drops in each well of a 24-well plate. The cell aggregates were cultivated to form organoids, according to a protocol published previously.^21^ The organoids were cultivated for two passages before seeding them into mucosoids using a method established previously.^17^ Briefly, 250,000 cells derived from antrum organoids were seeded in 200 μL culture medium into collagen-coated (A10644–0, 12.5–15 μg/cm;^2^ Gibco) Transwell inserts (PIHP01250; Millipore) placed in a 24-well plate at 37°C, 5% CO_2_ in a humidified incubator. The space between filter and well was filled with 400 μL culture medium (Table 3). At day 3 post seeding, the medium overlying the cells was removed from the well insert to start the air–liquid interface culture. Subsequently, the 500 μL medium below the filter was replaced twice a week. After 13 days, only half of the medium volume was changed regularly to maintain cell-secreted factors in the medium. Every 30–45 days, the mucosoids were expanded; top and bottom of inserts were washed three times with PBS, followed by 30–60 min of incubation with 0.05% trypsin/EDTA (25300; Thermo Scientific) on both sides of the filter. Cells were harvested, washed, and reseeded at 250,000 cells per new filter.

**Table 3.** Composition of the culture medium.

Name	Concentration	Manufacturer	Code
ADF	18,45% V/V	Thermo Fischer	12634
Conditioned Wnt3A-medium	50% V/V	Home made	
Condioned R-spondin1 medium	25% V/V	Home made	
HEPES	10 mM	Thermo Fischer	15630-056
Glutamax	1% V/V	Thermo Fischer	35050-087
B27	2% V/V	Thermo Fischer	17504044
N2	1% V/V	Thermo Fischer	17502048
Human epidermal growth factor (EGF)	20 ng/ml	Thermo Fischer	PHG0311
Human noggin (Peprotech)	150 ng/ml	Peprotech	120-10C-1000
Human fibroblast growth factor (FGF)-10	150 ng/ml	Peprotech	100-26-1000
Nicotinamide	10 mM	Sigma	N0636
Numan gastrin	10 nM	Sigma	G9145
A83–01	1 µM	Calbiochem	616454
Y-27632^a^	7,5 µM	Sigma	Y0503

Note: ^a^after the 3rd day the concentration is reduced to 1,5 µM.

### Microarray analysis

Microarray experiments were carried out as single-color hybridizations using custom whole genome human 8 × 60k Agilent arrays (Design ID 048908), in accordance with the manufacturer’s instructions. Probe intensities were extracted using Agilent Feature Extraction software. The resulting single-color raw data files were analyzed using R v.4.4.2 and Bioconductor package limma v.3.56.2^22^ applying background correction with method “normexp” with offset 15 and quantile normalization. Differential gene expression analysis was performed using lmFit and eBayes functions from limma package accounting for the batch effect due by patient interindividual differences adding a covariate for patients in the regression model. Benjamini-Hochberg adjusted and nominal p-values were generated for each probe. Comparisons of microarray gene expression between groups were conducted using paired tests under different conditions. R packages ggplot2 v.3.5.0. and ComplexHeatmap v.2.16.0 were used to generate heatmaps. Broad Institute gene set enrichment analysis (GSEA)^23^ was used to assess the enrichment of the ranked gene expression profiles versus the curated “Hallmark” and selected C2 (REACTOME subcategory) and C5 (Gene Ontology, Biological Process subcategory) gene set collections from the Broad Molecular Signatures Database (MSigDB) v.7.5.1. Normalized enrichment score (NES) was also calculated with GSEA, by considering differences in pathway size (i.e., gene set size) and allowing for comparisons between the gene sets. The microarray data have been made publicly available in the Gene Expression Omnibus (GEO; https://www.ncbi.nlm.nih.gov/geo/query/acc.cgi?acc=GSE268591) database of the National Center for Biotechnology Information. These data can be accessed using the GEO accession number: GSE268591.

### Mass spectrometry sample preparation

The mucus samples were processed using the filter-aided sample preparation method^24^, with modifications as described by Rodriguez-Pineiro et al.^25^ Each sample was diluted in 200 μL of 6 M guanidinium hydrochloride in 0.1 M Tris/HCl, pH 8.5 (GuHCl), and supplemented with 5 non-human proteins (10 pmol/protein) as internal standards. Cysteines were reduced by adding 30 μL of 0.1 M dithiothreitol and incubating the mixture at 60°C for 20 min. The samples were then transferred to Microcon-30 kDa centrifugal filters (MRCF0R030) and washed with 200 μL of GuHCl. Alkylation was carried out by adding 100 μL of 0.05 M iodoacetamide and incubating the samples at room temperature for 20 min in the dark. This was followed by two washes with 100 μL of GuHCl and two more with 100 μL of 50 mM ammonium bicarbonate/5% acetonitrile. Proteins were digested overnight at 37°C with 0.2 μg of sequencing-grade modified trypsin (V5111; Promega) in 40 μL of 50 mM ammonium bicarbonate/5% acetonitrile. The resulting peptide mixtures were acidified with trifluoroacetic acid to a final concentration of 0.5% (vol/vol), desalted using ZipTip C18 (Millipore, 0.6-μL bed volume), and then lyophilized.

### Liquid chromatography with tandem Mass spectrometry analysis

The samples were analyzed using a QExactive Plus mass spectrometer (Thermo Fisher Scientific) connected to a Dionex UltiMate 3000 RSLCnano System (Thermo Fisher Scientific). Each sample was resolubilized in 13 μL of 2:98 (v/v) acetonitrile/water with 0.1% trifluoroacetic acid. Then, 10 μL was loaded onto a C18 PepMap 100 trap column (300 μm × 5 mm; 5 μm particle size, 100 Å pore size; Thermo Fisher Scientific). The flow rate was set at 20 μL/min using 2:98 (v/v) acetonitrile/water with 0.1% trifluoroacetic acid for preconcentration and desalting. Separation occurred on an Acclaim C18 PepMap RSLC column (75 μm × 250 mm; 2-μm particle size, 100-Å pore size; Thermo Fisher Scientific) at a flow rate of 300 nL/min. High-performance liquid chromatography solvent A (0.1% v/v formic acid in water) was used, and peptides were eluted with solvent B (80:20 v/v acetonitrile/water with 0.1% formic acid), starting from 3%, increasing to 40% over 85 min, and then to 98% in 5 min. The peptides were analyzed in a data-dependent acquisition mode, alternating between one mass spectrometry scan and ten tandem mass spectrometry scans for the most abundant precursor ions. The mass spectrometry scans covered a range of m/z 350–1600, with a resolution set to 70,000. Peptides were fragmented using higher-energy collisional dissociation at 27% normalized collision energy and measured in the orbitrap at a resolution of 17,500.

### Proteins identification

Proteins were identified and quantified using MaxQuant software (version 1.626), searching the SwissProt human sequence database (released 2018_11, containing 20,412 entries). The search parameters included a maximum of two missed cleavages, with variable modifications of Oxidation (M), Acetyl (Protein N-term), pyro-Glu (Gln), and carbamidomethylation of cysteines as a fixed modification. The false discovery rate was set to 0.01 for proteins, peptides, and modified sites. A comprehensive list of proteins identified under each condition is provided in Supplementary File 1.

### Western Blot analysis.

Mucus from mucosoids was collected and diluted with Laemmli buffer final concentration 2×. The samples were then sonicated and boiled for 10 min at 95°C. Proteins were separated by SDS-PAGE in 18% or 10% polyacrylamide gel according to the proteins molecular weight. After separation by electrophoresis, transfer was performed using Criterion blotter (Biorad). The Nitrocellulose membranes (Amersham Protran 0.45 μm) were stained 5 min with red ponceau (Sigma) and washed with Tris-buffered saline (TBS)-Tween 1X. After 1 h blocking with a 5% solution of fat-free milk in TBS-Tween 1X, the membranes were incubated with the following primary antibodies overnight at 4°C (Table 4). After 3 × 10 min washes, the membranes were developed using the corresponding secondary antibodies (Table 4). The antibody signal was detected with enhanced chemiluminescence substrate according to the manufacturer's instruction (Amersham ECL Select). Chemidoc MP imaging system (Biorad) was used for images acquisition.

**Table 4.** Antibodies.

Antibody	Host	Dilution IF	Dilution WB	Manufacturer	Code
C3a	Goat	1:100	1:1000	R&D systems	AF3677
CXCL9	Rabbit	1:100	1:1000	Cell signaling	30327S
Lipocalin	Mouse	1:200	1:1000	Abcam	ab23477
Lactotransferrin	Mouse	1:100	1:200	Santa Cruz	sc -53,498
Anti-Mouse AF546	Donkey	1:500	N/A	Invitrogen	A10036
Anti-Rabbit AF488	Goat	1:500	N/A	Invitrogen	A11008
Anti-Goat AF647	Chicken	1:500	N/A	Invitrogen	A21449
Anti-Rabbit HRP	Goat	N/A	1:10000	Biorad	STAR208P
Anti-Mouse HRP	Goat	N/A	1:10000	Biorad	STAR208P
Anti-Goat HRP	Rabbit	N/A	1:1000	Biorad	1721034
Anti CagA – B300	Rabbit	1:100	N/A	Santa Cruz	sc -25,766
Anti CagA BK-20	Goat	1:100	N/A	Santa Cruz	sc -48,128

### RNA isolation and quantitative real-time polymerase chain reaction analysis

Filters containing the cells of the mucosoid cultures were excised from the inserts and total RNA was extracted and purified using the RNeasy Mini Kit (Qiagen, Hilden, Germany), according to the manufacturer’s specification. The total RNA concentration was quantified using a NanoDrop spectrophotometer. For reverse transcription, the Tetro cDNA Synthesis Kit (Bioline) was employed. Quantitative real-time polymerase chain reaction (qPCR) was performed using the QuantStudio3 Real-Time PCR System (ThermoFisher) and the SensiMix SYBR low-ROX Kit (Bioline). The specific primers used in this study are detailed in Table 5. Results from three independent experiments were analyzed using the Delta-Delta Ct method. HPRT1 was used as a human reference gene.

**Table 5.** Primers.

Gene		Sequence
HPRT1	Fw	5’-GAC CAG TCA ACA GGG ACA *T*-3’
	Rv	5’-CCT GAC CAA GGA AAG CAA AG-3’
IL8	Fw	5’-ACA CTG CGC CAA CAC AGA AAT-3’
	Rv	5’-ATT GCA TCT GGC AAC CCT ACA-3’
CXCL9	Fw	5’-ATT GGA GTG CAA GGA ACC CC-3’
	Rv	5’-GGG CTT GGG GCA AAT TGT TT-3’
LTF	Fw	5’-TGC TCC ACC AAC AGG CTA AA-3’
	Rv	5’-GCG ACA TAC TGT GGT CCC AA-3’
C3	Fw	5’-GCG AAT GGA CAA AGT CGG C-3’
	Rv	5’-CTC TGG GAA CTC ACT TCG GG-3’
NGAL	Fw	5’-ACT TCC AGG ACA ACC AAT TCC AG-3’
	Rv	5’-GGC AAC CTG GAA CAA AAG TCC-3’
TNFα	Fw	5’-TGA AAG CAT GAT CCG GGA CG-3’
	Rv	5’-CAG CTT GAG GGT TTG CTA CAA C-3’
IFNγ	Fw	5’-TCGGTAACTGACTTGAATGTCCA-3’
	Rv	5’-TCGCTTCCCTCTTTTAGCTGC-3’
IL-1β	Fw	5’-ATGATGGCTTATTACAGTGGCAA-3’
	Rv	5’-GTCGGAGATTCGTAGCTGGA-3’
rDNA 16s *H. pylori*	Fw	5’-TTTGTTAGAGAAGATAATGACGGTATCTAAC-3’
	Rv	5’-CATAGGATTTCACACCTGACTGACTAT C-3’
gDNA hGAPDH	Fw	5’-GACTTCAACAGCGACACC C-3’
	Rv	5’-AGAAGATGAAAAGAGTTGTCAGGGC-3’

### *Cultivation of* H.pylori

*H.pylori*-GFP is a P12 isogenic fluorescent strain, resistant to kanamycin. The cultivation was done in agar plates containing 36 g/L GC agar base (Remel; supplied by Oxoid, R453502) supplemented with 10% inactivated horse serum (Biochrom, S 9135), 1% nutrient mix (100 g/L D(+)Glucose, 10 g/L, L-Glutamin), 26 g/L, 100 mg/L vitamin B1, 20 mg/L Iron(III) nitrate ninhydrate, 3 ml/L Thiamine hydrocloide, 13 mg/L 4 aminobenzoic acid, 250 mg/L nicodinamide adenine dinucleotide, 10 mg/L Vitamin B12, 1.1 g/L L-Cysteine, 1 g/L Adenine hemisulfate, 30 mg/L Guanine Hydrochloride, 150 mg/L L-Arginine monohydrochloride, 500 mg/L Uracil, 0,48% HCL in water), 1 μg/mL Nystatin, 5 μg/mL Trimethoprim, 10 μg/mL Vancomycin and 8 mg/mL Kanamycin.

### Bacterial survival assay in the mucus

Single epithelial cells from three different mucosoid lines were seeded onto new transwells for 15 days prior to starting the different treatments. Mucus was harvested after the treatments and centrifuged at 1700×g at room temperature for 4 min to remove debris. A suspension of 5 μL with 10^5^ CFU of a kanamycin resistant P12 isogenic *H. pylori* GFP strain was resuspended in 20 μL of the harvested mucus samples or in PBS as control and incubated for 120 min in a microcentrifuge tube at 37°C. 20 μL of four serial dilutions (10^−1^ – 10^−4^) were plated on supplemented GC agar plates. Plating of the suspension was performed by pouring the liquid on the edge and inclining the plate in one direction to spread the liquid in line without touching the skirt of the plate. Plates were incubated at 37°C, 5% CO_2_ and 5% O_2_, face-up for 1 h to let the excess liquid evaporate and then turned upside down for 3 days. Colonies were counted and results are presented as the percentage of colonies relative total colonies present in PBS controls (Figure 3(f)).

### Bacterial survival assay in a solution with synthetic antimicrobials

10^5^ CFU of *H. pylori* P12-GFP (kanamycin resistant) were incubated for 60 and 120 min in a solution of 25 μL of PBS containing 5, 10 or 30 μg/ul of antimicrobial peptide (Table 6). Each AMP was tested individually, three times at each concentration. The suspension was plated in supplemented GC agar plates as it was done for the bactericidal assay of the mucus. The results are presented as the percentage of CFU relative to the one in PBS.

**Table 6.** Sequence of the AMPs.

**hLFcin** GRRRRSVQWC^10^ AVSQPEATKC^20^ FQWQRNMRKV^30^ RGPPVSCIKR^40^ DSPIQCIQA^50^
**hLCN2** MPLGLLWLGL^10^ ALLGALHAQA^20^ QDSTSDLIPA^30^ PPLSKVPLQQ^40^ NFQDNQFQGK^50^ WYVVGLAGNA^60^ ILREDKDPQK^70^ MYATIYELKE^80^ DKSYNVTSVL^90^ FRKKKCDYWI^100^ RTFVPGCQPG^110^ EFTLGNIKSY^120^ PGLTSYLVRV^130^ VSTNYNQHAM^140^ VFFKKVSQNR^150^ EYFKITLYGR^160^ TKELTSELKE^170^ NFIRFSKSLG^180^ LPENHIVFPV^190^ PIDQCIDG
**hC3a** SVQLTEKRMD^10^ KVGKYPKELR^20^ KCCEDGMREN^30^ PMRFSCQRRT^40^ RFISLGEACK^50^ KVFLDCCNYI^60^ TELRRQHARA^70^ SHLGLAR
**hCXCL9** TPVVRKGRCS^10^ CISTNQGTIH^20^ LQSLKDLKQF^30^ APSPSCEKIE^40^ IIATLKNGVQ^50^ TCLNPDSADV^60^ KELIKKWEKQ^70^ VSQKKKQKNG^80^ KKHQKKKVLK^90^ VRKSQRSRQK KTT

### *Mucosoid treatment with cytokines and treatment with cytokines in combination with* H.pylori *infection*

Three weeks post-seeding, 5 ng/mL TNFα, 2.5 ng/mL IL1β and 10 ng/mL IFNγ were added individually or in combination in the culture medium of mucosoids for 5 days. In case of concomitant infection, 2 days after cytokines combination treatment, a suspension of 30 μl of *H.pylori* (MOI 100) was added on the mucus of the mucosoids for the remaining 3 days (Figure 5(a)). To calculate the MOI, the number of cells (usually 600.000 to 700.000) was calculated from an identical mucosoid culture.

### Immunofluorescence staining of mucosoids

Mucosoids on the filters were fixed in 4% paraformaldehyde for 20 min at room temperature and washed with PBS. The filters were incubated with blocking solution (PBS, 5% donkey serum, 0.3% Triton X-100) for 1 h, followed by primary antibody in antibody dilution solution (PBS, 1% BSA + 0.3% triton X-100) overnight at 4°C. After three washes with PBS, samples were incubated with fluorescently labeled secondary antibodies and 4′,6-diamidino-2-phenylindole (1:1000) for 2 h at room temperature in the dark. The antibodies and the relative dilutions are listed in Table 4 Samples were washed three times with PBS, mounted in Mowiol, and analyzed by confocal microscopy using a Zeiss 980 microscope. Images were processed and analyzed with ImageJ.

### Software

Data analysis and plot generation were performed using R, MaxQuant, Microsoft Excel, and GraphPad Prism. Figures were assembled in Adobe Illustrator, with schematics created via Biorender.com. The manuscript was written in Microsoft Word and edited with the assistance of ChatGPT 4 to correct grammar and spelling.

## Results

### The effect of pro-inflammatory cytokines on gastric epithelial cells

**Figure 1.**
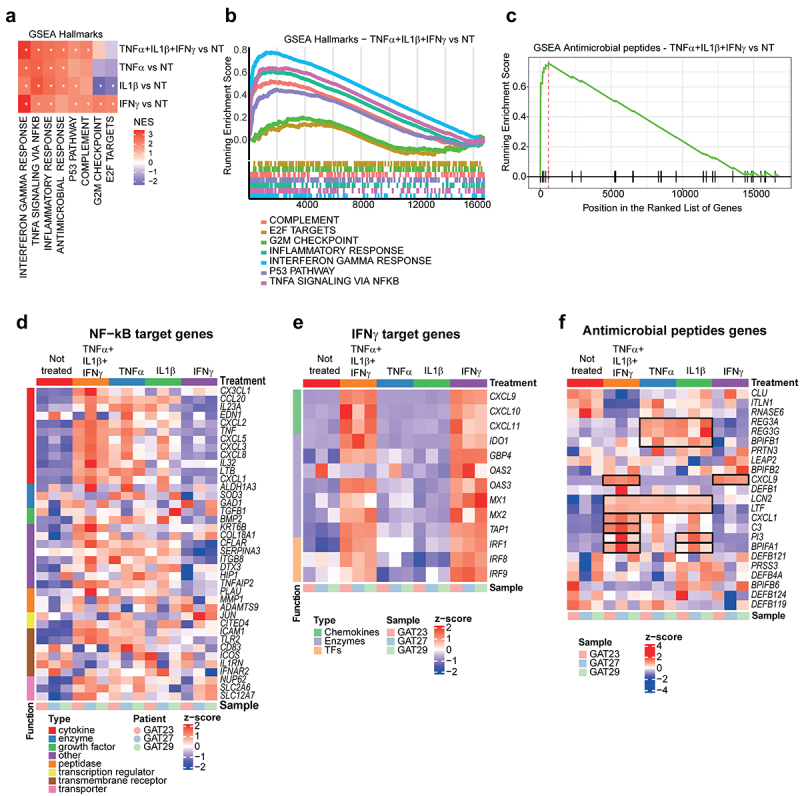
Inflammation triggers the expression of genes encoding for antimicrobial peptides. Cells from three different mucosoid lines were treated with TNFα (5 ng/mL), IL1β (2.5 ng/mL), and IFNγ (10 ng/mL) combined or individually (as indicated), for 5 days before extracting RNA for transcriptomic profile by microarray. (a). Heatmap of the most relevant GSEA hallmarks (https://www.gsea-msigdb.org/gsea/msigdb/collections.jsp) relative to the comparison of the transcriptome of the treated vs. untreated mucosoids. NES= normalized enrichment score, *= false discovery rate < 0.0001. Red = upregulation, white = no change, blue = downregulation. (b). Enrichment plot of the most significant GSEA hallmark resulting from the comparison of the transcriptome of the mucosoids treated with TNFα, IL1β, and IFNγ, compared to the untreated samples. (c). Enrichment plot of the GSEA hallmark “antimicrobial peptides” (d). Heatmap of validated nf-κB target genes as reported in (https://www.bu.edu/nf-kb/gene-resources/target-genes/) (e). Heatmap of selected interferon-dependent chemokines, enzymes and transcription factors. (f). Heatmap of genes coding for antimicrobial peptides. Genes showing consistent upregulation in at least one of the conditions are outlined in black.

Epithelial cells were isolated from the antrum of healthy stomachs derived from patients having undergone weight loss surgery (Table 1). Cells were cultivated on porous transwells at the air-liquid interface with a supplementation of growth factors to generate mucosoid cultures (Table 3).^17^ As previously observed, the inclusion of WNT3A, RSPO1, EGF and NOGGIN in the culture cocktail induced a mucus neck cells phenotype^17,19,26^ and the secretion of a MUC6 enriched mucus to the apical side.^17^ To study how epithelial cells respond to inflammatory stimuli, we analyzed the transcriptome of gastric mucosoids treated with TNFα, IL1β and IFNγ in combination or single cytokine treatment. These cytokines are commonly found in the inflamed gastric mucosa.^27–29^ Analysis of the genes upregulated in cytokine treated mucosoids revealed that pro-inflammatory pathways are significantly enriched (Figure 1(a)). We also find a positive correlation with genes encoding for AMPs (Figure 1(c)). Single treatment with TNFα and with IL1β revealed a similar gene enrichment pattern and positive regulation of antimicrobial related genes (Supplementary SFig.1A-D). This similarity might be explained by the fact that both TNFα and IL1β activate the NF-κB pathway, a major effector of inflammation (Figure 1(d)). However, the combination of TNFα, IL1β and IFNγ induces a stronger NF-κB response (Figure 1(d) and Supplementary SFig.2A). Treatment with IFNγ specifically induced IFNγ target genes (Figure 1(e), Supplementary SFig.1E and Supplementary SFig.2B) but caused milder enrichment in antimicrobial peptide-associated genes compared to mucosoids treated with TNFα or IL1β (Supplementary SFig.1F). Among all possible expressed AMPs in the mucosoids (Figure 1(f )), we found *LCN2* (Licopalin2), *LTF* (lactotransferrin), *CXCL1* (C-X-C motif ligand 1), *C3* (complement component 3) and *BPIFA1* (BPI fold containing family A member 1) to be consistently up-regulated after treatment with TNFα, IL1β and IFNγ *REG3A* and *REG3G* (human regenerating islet-derived protein 3-alpha and gamma), and *BPIFB1* (BPI fold containing family B member 1) expression was found to be dependent on IL1β and TNFα. *CXCL9* (C-X-C motif ligand 1) was instead regulated only in the presence of IFNγ. Other antimicrobials previously reported in the stomach or in stomach cells^14,30,31^ such as defensins (e.g., DEFB4A, DEFB1, DEFB124, DEFB121 and DEFB103) or Cathelicidins (e.g., LL-37) did not undergo significant changes in their expression, or were undetectable, when the epithelium is inflamed. This suggests that other non-epithelial cells may be the source of these antimicrobials, or that their regulation does not depend on TNFα, IL1β or IFNγ.

### Inflammatory signals stimulate the epithelium to secrete antimicrobials in the mucus

**Figure 2.**
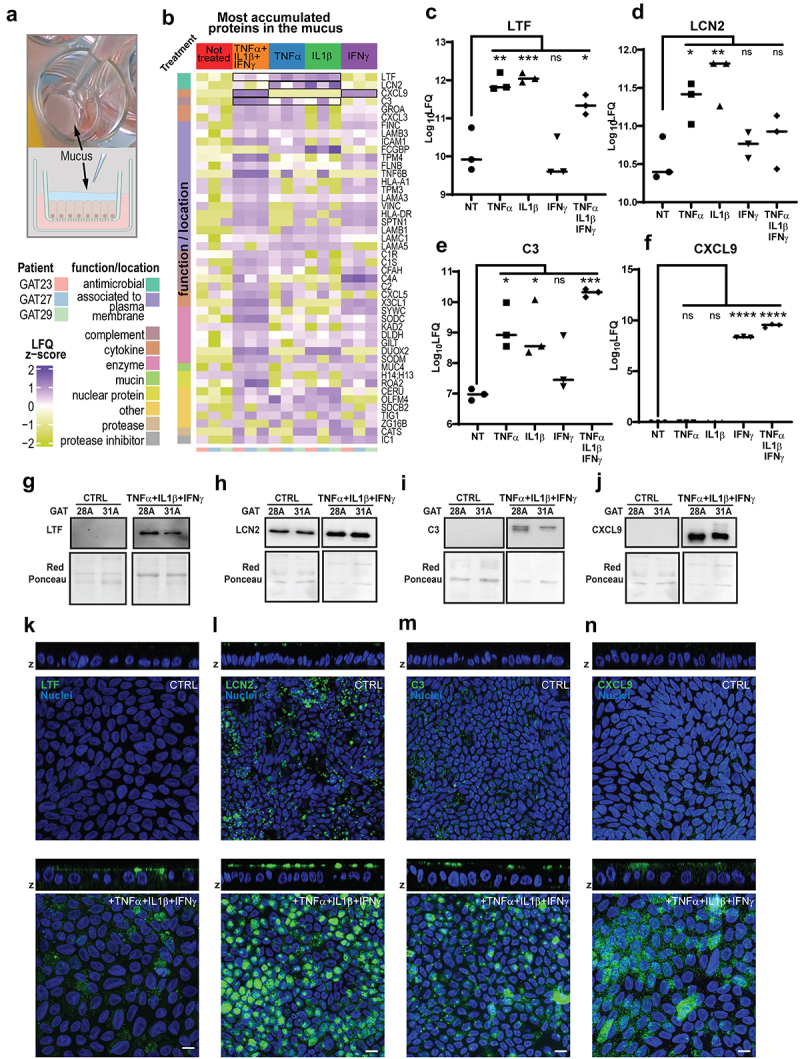
Inflammation induce the secretion of AMPs in the mucus. (a). Mucus was removed from three different mucosoid lines after treatment with TNFα (5 ng/mL), IL1β (2.5 ng/mL), and IFNγ (10 ng/mL) combined or individually (as indicated) for 5 days. The total mucus secreted and accumulated was analyzed by mass spectrometry. (b). Heatmap of the abundance of the proteins in the mucus determined by label free quantification (LFQ). Only proteins with an altered expression of at least 10-fold in one of the conditions are represented. Outlined in black are those proteins with reported bactericidal activity. The other proteins are categorized by location or function. (c)(d)(e)(f) The Log_10_LFQ values are plotted for LTF (lactotransferrin), LCN2 (lipocalin 2), C3 (complement component C3), CXCL9 (C-X-C motif chemokine ligand 9) the horizontal bar represent the median of tree individual measurements from the mucus produced by three different mucosoid lines (GAT23, 27, 29). Multiple comparison test was performed after one way ANOVA to assess if the differences between treated and untreated samples are significant: *p = <0.05, **p = <0.005, ***p = <0.0005, **** *p*= <0.00005. The relative abundance of other antimicrobials CXCL1 and CXCL3 did not pass the statistical test for any of the multiple comparisons. (g)(h)(i)(j) validation of expression of the antimicrobial was confirmed by Western blot using a further two samples of mucosoids (GAT28, GAT31) treated with the combination of pro-inflammatory cytokines as before. As housekeeping proteins are not yet available for the mucus, Red Ponceau was used as a loading control. (k)(l)(m)(n) Mucosoids were treated with a combination of pro-inflammatory cytokines as before, and immunofluorescence experiments carried out to localize the expression of intracellular LTF, LCN2, C3 and CXCL9. Stained whole mount mucosoids were imaged using confocal microscopy. Images were taken across the whole thickness of the monolayer (z-stacks) and the panels shows a top and a lateral projection. Scale bar = 5 µm.

To understand if the proinflammatory signals TNFα, IL1β and IFNγ regulate epithelial secretions to the mucus, we analyzed by mass spectrometry the total protein content of the mucus accumulated for five days (Figure 2(a)) in inflamed *vs* non inflamed mucosoids. We found numerous proteins in the mucus, such as mucins, proteases, enzymes, and cytokines, (Supplementary file 1) however, only some of these were differentially expressed between inflamed and non-inflamed gastric epithelial cells (Figure 2(b) and Supplementary File 2). We also observed plasma membrane proteins as well as proteins from other intracellular compartments, suggesting that intracellular content, probably resulting from dead cells, could be found in the mucus. Four known AMPs are significantly differentially accumulated in the mucus of the inflamed epithelium: Lactotransferrin (LTF), Lipocalin2 (LCN2), Complement component 3 (C3) and C-X-C motif chemokine 9 (CXCL9) (Figure 2(c–f)). LTF is an antimicrobial found in milk and other body secretions.^32^ LCN2 is a known major antimicrobial player in gut immunity.^33^ C3 is a subunit of the complement system with antimicrobial activity.^34^ CXCL9 is an antimicrobial whose activity is independent from the chemokine function of this peptide.^35^

Other proteins encoded by AMP genes identified in the transcriptome analysis (Figure 1(f)) were either undetectable, or their variation in the mucus following inflammation in the epithelium was not statistically significant. The expression of LTF, LCN2, C3 and CXCL9, was confirmed by Western blot analysis of the mucus (Figure 2 (g–k)) and their intracellular expression localized at the apical side of the mucosoids, consistent with the fact that these proteins are secreted in the mucus (Figure 2, (k–n)).

### *The efficacy of epithelial antimicrobials against* Helicobacter pylori

**Figure 3.**
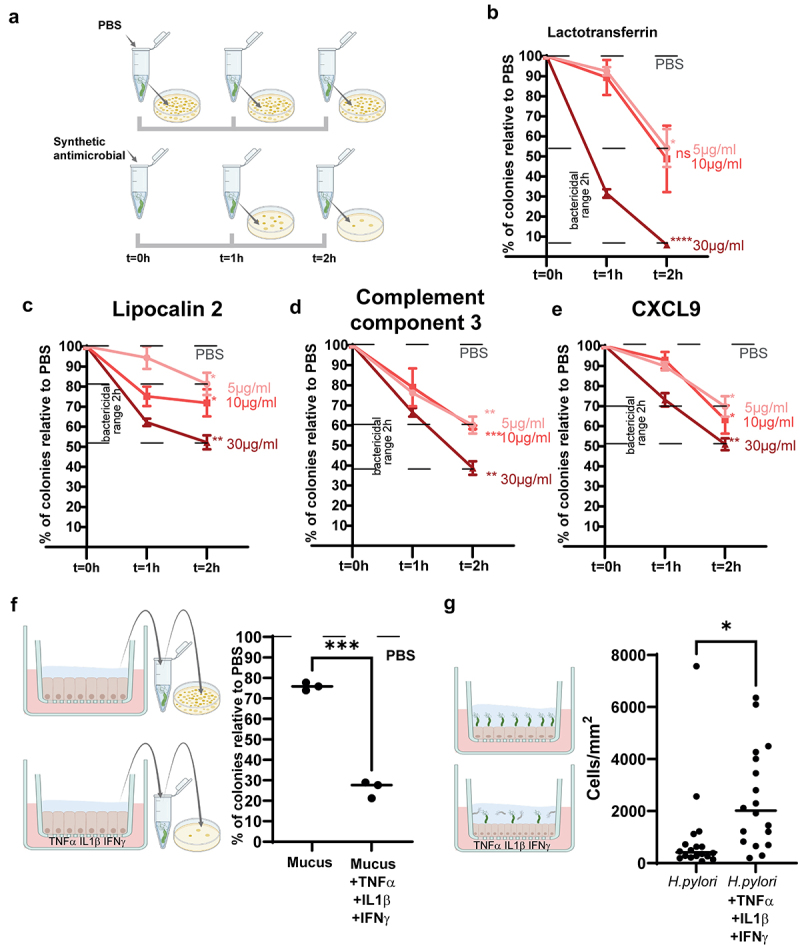
The bactericidal activity of antimicrobial peptides found in the mucus. ((a) A suspension of 10^5^ CFU of kanamycin resistant *H.pylori* was incubated for 1 h and 2 h in the presence of different concentrations (5, 10, or 30 mg/mL) of AMPs. The suspension was plated in kanamycin plates and the number of colonies was counted relative to PBS to assess the bactericidal range of each antimicrobial. (b). Bactericidal activity of Lactotransferrin using the bioactive peptide lactoferricin (LFcin), the antimicrobial peptide derived from the hydrolysis of lactotransferrin. (c). Bactericidal activity of LCN2. (d). Bactericidal activity of C3a, the peptide derived from complement component 3. (e). Bactericidal activity of CXCL9. Multiple comparison test was performed after a two way ANOVA to assess if the difference in bactericidal activity, for each concentration of AMP, between different time points, is statistically significant. *p* values comparing t = 2 h and t = 0 are reported. ns= non significant. *p = <0.05, **p = <0.005, ***p = <0.0005, ****p = <0.00005. (f). Mucosoids were treated with TNFα (5 ng/mL), IL1β (2.5 ng/mL), and IFNγ (10 ng/mL) for 5 days, with a fresh supply of cytokines added on the third day. Mucus samples from mucosoids derived from 3 different patients was incubated with 10^5^ CFU of a kanamycin resistant isogenic P12 strain for 2 hours before plating and assessing the bactericidal activity by counting the percentage of colonies relative to PBS. Paired samples t-test was used to assess if the difference between the bactericidal activity of the two groups was significant. ***= p < 0.0005. (g) Mucosoids were treated with a combination of pro-inflammatory cytokines as before and infected on the second day with *H.pylori* at MOI 100 for 3 days. Mucosoids were washed with PBS to remove the dead cells, they were stained with DAPI and imaged using confocal microscopy to count the remaining cells after infection. Each dot represents the density of cells per area of imaging (0.18 mm^2^) and paired samples t-test was used to assess if the difference between the groups is significant *p=<0.05.

To test if the AMPs found in the mucus were active against *H. pylori in vivo*, we incubated *H. pylori* with commercially available recombinant versions of the antimicrobial domains of these proteins at a range of concentrations (5–30 μg mL^−1^) for 1 h and 2 h, and then selectively plated these solutions to assess bactericidal activity based upon the number of Colony Forming Units (CFUs) relative to a PBS control (Figure 3(a)). The 49 amino acid bioactive portion of human Lactotransferrin^36^ displayed the greatest activity against *H.pylor*i, with a 90% CFU reduction in 2 h using the concentration of 30 μg/mL of the peptide (Figure 3(b)). Lipocalin2 showed a moderate bactericidal effect (20–50% of CFU reduction in 2 h) (Figure 3(c)). Moderate antimicrobial activity was also observed for the recombinant C3 and CXCL9 (Figure 3(d, e)) (40%–60% and 30%–50% of CFU reduction in 2 h, respectively). These results demonstrate that, individually, the AMPs found in the mucus exert an antimicrobial activity against *H. pylori*. As these AMPs are secreted together in the mucus, we next investigated if native mucus from the inflamed epithelial cells of mucosoids shows antimicrobial activity. Mucosoids were treated again with TNFα, IL1β and IFNγ in combination, and mucus was harvested after 5 days of accumulation in treated and untreated controls. *H. pylori* was able to form significantly fewer colonies after incubation with mucus from inflamed cells, compared to mucus from non-inflamed cells (Figure 3(f)). To test if this mucus protects against infection, we then treated mucosoids with TNFα, IL1β and IFNγ for 5 days and concomitantly infected with *H.pylori* from day 2 to day 5 (Figure 3(g)). Our results demonstrate that there was a significantly higher number of remaining cells in the mucosoid cultures treated with pro-inflammatory cytokines compared to the non-treated (419 cells/mm^2^ vs. 2016 cells/mm^2^. This suggests that the mucus from inflamed cells conferred a partial protection against *H.pylori* infection.

### AMPs gene expression is increased in chronic gastritis

**Figure 4.**
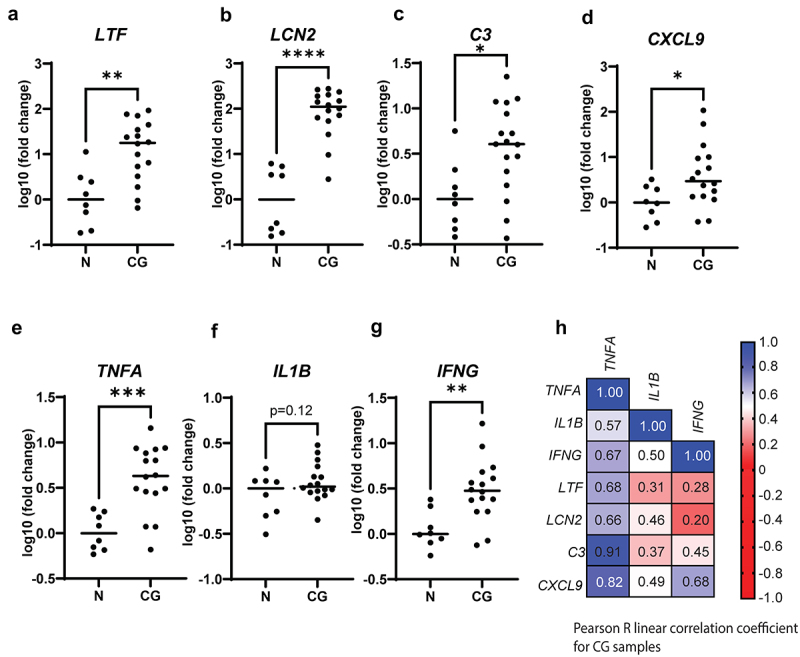
The expression of AMPs in biopsy samples from patients with chronic gastritis. RNA from 16 biopsy samples from *H.pylori* positive chronic gastritis (GC) cases and 8 from normal-looking stomach (N) was retrotranscribed into cDNA for the detection of AMP genes and of genes encoding for pro-inflammatory cytokines. (a)–(g). The Log10 relative expression of *LTF*, *LCN2*, *C3*, *CXCL9*, *TNFA*, *IL1B* and *IFNG* is reported. The significance of the difference between the groups was calculated using an unpaired t-test. *p=<0.05, **p=<0.005, ***p=<0.0005, ****p=<0.00005. (h). The Log10 fold induction value of each gene detected in the chronic gastritis biopsies was used to compute a pearson R linear correlation heatmap. 1 = highest correlation, 0 = no correlation, −1=highest negative correlation.

Chronic gastritis is an inflammatory condition of the stomach often caused by chronic *H. pylori* infection.^11^ Association with *H. pylori* infection can be made after the detection of the bacteria via urea breath test, histopathology report and *H. pylori* blood antibody test. As *Helicobacter pylori* can colonize deep within the glands^37,38^ where MUC6+ cells are located, we decided to test the hypothesis that AMPs found in inflamed mucosoids are upregulated in patients with *H. pylori-*related chronic gastritis. We analyzed 16 biopsies from *H.pylori*-positive chronic gastritis cases and 8 healthy unmatched controls from *H. pylori* negative patients without inflammation in the stomach (Table 2). We tested the expression of *LTF*, *LCN2*, *C3* and *CXCL9* by qPCR and found that these AMPs were significantly upregulated in the chronic gastritis group compared to healthy controls (Figure 4(a–d)). We also tested gene expression of the pro-inflammatory cytokines TNFα, IL1β and IFNγ Figure 4(e–g), and correlated this to the expression of the AMPs (Figure 4(h)). *TNFA* showed the strongest linear correlation (R Pearson coefficient) with all four antimicrobials. *IL1B* poorly correlated, while *IFNG* correlated only with CXCL9 (an interferon target gene). We also measured the relative amount of *H. pylori* DNA within the biopsies. As expected, there was a significant increase in *H. pylori* DNA in biopsies from patients with *H. pylori*-associated chronic gastritis (Supplementary SFig.3A). However, *H. pylori* DNA in biopsies did not correlate with the expression levels of genes encoding AMPs or those encoding pro-inflammatory cytokines (Supplementary Fig.3B). Altogether these results indicate that TNFα is likely to be the key factor for stimulating epithelial antimicrobial defense in the mucus.

### Helicobacter pylori *reduces the expression of epithelial antimicrobials*

**Figure 5.**
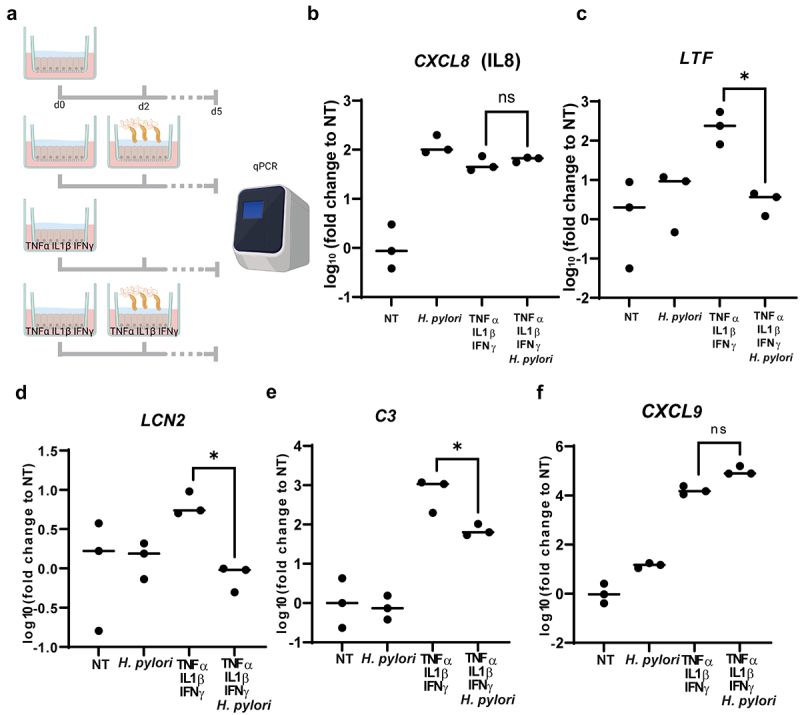
*H. pylori* downregulates the expression of antimicrobials of inflamed epithelial cells (a). Three different mucosoid lines were treated by four different protocols, according to the schematic. Upper: non-treated (NT); second row: *H. pylori* infection carried out at day 2 at MOI 100; third row: treatment with pro-inflammatory cytokines (TNFα, 5 ng/mL; IL1β, 2.5 ng/mL; IFNγ 10 ng/mL) from day 0; lower: treatment with pro-inflammatory cytokines at the same concentrations as previously from day 0, following *H. pylori* infection at MOI 100 at day 2. At day 5, RNA was extracted and the abundance of transcripts was measured relative to non-treated controls. Due to the large differences between the expression of genes under different treatments, results are expressed in Log10-fold change. (b). The expression of *CXCL8* does not change when *H. pylori* infection follows the treatment with pro-inflammatory cytokines. (c)(d)(e) The expression of *LTF, C3* and *LCN2* is downregulated when *H. pylori* infection is carried out in addition to the treatment with pro-inflammatory cytokines. (f) CXCL9 expression does not significantly change when *H. pylori* infection is in addition to to the treatment with pro-inflammatory cytokines.

*H. pylori* has the exceptional capacity to persist within the stomach mucosa for decades if not eradicated with antibiotics.^11^ While it is known that it can evade adaptive immunity, we now question how the bacteria survive given the presence of antimicrobials secreted by the inflamed epithelium. To investigate whether *H. pylori* is able to subvert host epithelial innate immune defense, we treated mucosoids with the same mixture of pro-inflammatory cytokines (TNFα IL1β IFNγ) that triggered the secretion of LTF, LCN2, C3 and CXCL9, and then infected them with *H. pylori* (Figure 5(a)). The level of expression of IL8 (*CXCL8*) remained high in case of exposure to cytokines followed by infection (Figure 5(b)), suggesting that the level of inflammation remains constant. However, we noticed that in cytokine-treated mucosoids, the following infection with *H. pylori* caused a reduced expression of *LTF*, *LCN2* and *C3* (Figure 5(c-e)). *CXCL9* expression was not significantly different whether *H. pylori* infection was present or not (Figure 5(f)). Interestingly, while *LTF, LCN2* and *C3* regulation depends on TNFα (Figure 2(c–e)) and correlates with *TNFA* expression in chronic gastritis patients (Figure 4(h)), CXCL9 is the only antimicrobial dependent on IFNγ (Figure 2(f)). As TNFα promotes NF-κB activation, *H. pylori* might mediate the downregulation of *LTF, LCN2* and *C3* by uncoupling TNFα or NF-κB pathways activation from the induction of the expression of the antimicrobials.

Multiple comparison test was performed after one way ANOVA to assess if the differences in gene expression between different treatments are significant. Only the result of the test comparing the samples treated with cytokines and the one infected and treated with cytokines are shown. ns = non significant. *p=<0.05.

## Discussion

The gastrointestinal mucosa serves as a crucial barrier in the human body, fulfilling both digestive functions and defensive roles against microbial invasion. When infected, this mucosa becomes inflamed and attracts cells from both the innate and adaptive immune systems. The roles of various immune cells in defense are well-recognized, while the main contribution attributed so far to the epithelium is to produce mucus, which forms a physical barrier that prevents the attack of pathogens. Any further active defensive role of the epithelium remains to be demonstrated.

Mucus studies depend on the availability of this body fluid. Mucus can either be prepared directly from fixed animal or human tissue^39^ or harvested from tissues mounted in perfusion chambers.^25,40^ These approaches, although capable of providing in-depth analysis of mucus under native conditions, have limited experimental settings for testing variations in mucus secretion. Alternatively, mucus-producing cell lines can be grown in polarized monolayers, a method commonly employed for airway

epithelium mucus.^41^ However, as these cell lines often originate from cancerous tissues, their signaling pathways are altered, and their capacity to produce a robust mucus layer is generally reduced. Therefore, the regulation of protein secretion in the mucus remains poorly studied.

Bridging the gap between animal models and cell lines, organoid technology offers an unprecedented opportunity to explore mucus biology in depth.^42^ By transforming spherical organoids into mucosoid cultures we obtained the formation of an epithelial barrier that accumulates mucus on the apical side^17,43^ while retaining the stem cell-driven regeneration and multilineage differentiation capacity typical of the organoids. Mucosoid cultures, developed from healthy human stomach tissue, allow us to investigate for the first time how mucus secretion responds to various experimental conditions. In this study, we focused on antimicrobial peptides as they are recognized innate immune weapons against infection, known to be present in the mucus of various enteric^5,44,45^ and non-enteric mucosae.^46^

We found that inflammation induces the stomach epithelium to secrete antimicrobial peptides in the mucus. Specifically, we observed that TNFα, IL1β and IFNγ induce the secretion of four antimicrobials in the mucus: Lactotransferrin, Lipocalin2, complement component 3, and CXCL9. The mucus from inflamed cells and these specific antimicrobials have a significant anti-*H. pylori* activity *in-vitro* and the expression of these antimicrobials was also confirmed in biopsies from patients with inflammation in the stomach. Finally, we find that *H. pylori* counteracts the inflammation-induced expression of Lactotransferrin, Lipocalin2 and complement component 3.

Lactotransferrin (or Lactoferrin, LTF, TRLF, Lf) is an iron binding protein found at different concentrations (from 1–3 µg/mL to 8 mg/mL) in different body fluids including breast milk, colostrum, tears, and saliva.^47–49^ Its antimicrobial activity is independent from iron and is rather attributed to a peptide, called lactoferricin (Lfcin), derived from its hydrolysis or digestion with pepsin in the stomach. The antimicrobial properties of lactoferricin depend on the number of positively charged arginine residues located at the N-terminal domain.^50,51^ These residues can bind and disrupt the membranes of bacteria.^50^ In some countries, lactotransferrin is a commercially available prebiotic derivation from cow’s milk. However, while human Lfcin contains four arginine residues, the bovine variant has only two. This makes human Lfcin binding stronger to bacterial LPS compared to the bovine version.^51^ Notably, human lactotransferrin has demonstrated effectiveness in controlling *H. pylori* growth both *in vitro*^52^ and in animal models.^53^ Here, we demonstrate that this antimicrobial is directly produced by inflamed human stomach epithelial cells.

Lipocalin2 (or NGAL) is a major player in gut immunity by maintaining bacterial populations far from the mucosa.^33^ The bactericidal capacity depends on its ability to bind bacterial siderophores which prevent bacterial uptake of iron.^54^ C3 is a subunit of the complement system with antimicrobial

activity. In particular, C3a is the peptide that results from the cleavage mediated by C3 convertase or by spontaneous hydrolysis of C3 in water. This peptide has a known antimicrobial activity from invertebrates to humans and it is also referred to as anaphylatoxin. The bactericidal activity depends on the capacity of this peptide to permeabilise the bacterial membrane.^55^ CXCL9 is a chemokine but its chemokine-independent antimicrobial activity was reported against *Citrobacter rodentium* infection in the gut.^34,56^ The bactericidal mechanism is currently unknown. The different mode of action of these antimicrobials implies that the human stomach epithelium has evolved multiple strategies to prevent bacterial colonization.

However, we also noted that while inflammation typically increases the expression of AMPs, the presence of *H. pylori* infection leads to a decrease in their expression. This observation suggests that *H. pylori* has developed mechanisms to circumvent epithelial innate defenses, aligning with its exceptional colonization ability. In the context of chronic infections, it can be hypothesized that if *H. pylori* rapidly colonizes a new gland before it is eliminated by the mucus, the bacterium may neutralize the antimicrobial secretions of the epithelium, thereby facilitating the establishment of a new colony in a protected niche. Interestingly, *H. pylori* particularly reduces the expression of Lactotransferrin, Lipocalin2, and C3, which depend mainly on TNFα for their production. This indicates that *H. pylori* uncouples the pro-inflammatory signal of TNFα from its antimicrobial effectors. Interestingly both TNFα^57^ as well as *H. pylori*^58^ can elicit an inflammatory response in epithelial cells through the activation of NF-κB. However, the pathways of activation differ; *H. pylori* mediates p65 nuclear translocation via the activation of the kinase ALPK1.^59–61^ It is currently unknown whether the ALPK1-mediated activation of NF-κB is synergistic with, or acts in opposition to, the TNFα pathway for specific target genes. Moreover, the co-transcription factors involved in the expression of the antimicrobials found in this study are still unknown and merit further investigations. Understanding this dysregulation could reveal the key signaling molecules used by the bacterium and inform a potential pharmacological approach to increase the secretion of these AMPs, providing support in eradicating the bacteria. Potential host-directed approaches to restore AMP expression in infected tissues could represent an attractive therapeutic avenue, especially in scenarios where antibiotics are becoming less effective.

## Abbreviations

AMPs: anti microbial peptides
BPIFA1: BPI fold containing family A member 1
BPIFB1: BPI fold containing family B member 1
BSA: bovine serum albumin
C3: complement component 3
cDNA: complementary deoxyribonucleic acid
CFU: colony forming unit
CG: chronic gastritis
CXCL1: C-X-C motif ligand 1
CXCL8: C-X-C motif ligand 8
CXCL9: C-X-C motif ligand 9
DEFB 4A,1,124,121,103: beta defensins 4A,1,124,121,103
ECL: Enhanced chemiluminescence
EDTA: Ethylenediaminetetraacetic acid
EGF: epidermal growth factor
GFP: gene set enrichment analysis
HPRT1: hypoxanthine phosphoribosyltransferase 1
IFNγ: interferon gamma
IgM, IgG: immunoglobulin M, G
IL1β: interleukin 1 beta
LCN2: Licopalin2
LL-37: cathelicidin antimicrobial peptide
LTF: lactotransferrin
MOI: multiplicity of infection
MUC6: mucin 6
NES: normalized enrichment score
NF-κB: Nuclear factor kappa-light-chain-enhancer of activated B cells
PBS: phosphate buffered saline
qPCR: quantitative real-time polymerase chain reaction
REG3A,G: human regenerating islet-derived protein 3-alpha and gamma
RSLC: Rapid Separation Liquid Chromatography
RSPO1: R-spondin-1
SYBR: N’,N’-dimethyl-N-[4-[(E)-(3-methyl-1,3-benzothiazol-2-ylidene)methyl]-1-phenylquinolin-1-ium-2-yl]-N-propylpropane-1,3-diamine
TBS: Tris-buffered saline
TNFα: Tumor necrosis factor Alpha
WNT3A: Wnt family member 3A

## Supplementary Material

Supplemental Material

## Data Availability

Transcriptomic data have been made publicly available in the Gene Expression Omnibus (GEO; https://www.ncbi.nlm.nih.gov/geo/query/acc.cgi?acc=GSE268591) database of the National Center for Biotechnology Information. These data can be accessed using the GEO accession number: GSE268591.
