# Supplementary material for: Inflammation promotes stomach epithelial defense by stimulating the secretion of antimicrobial peptides in the mucus
Source: Gut Microbes. 2024 Sep 8;16(1):2390680. doi: 10.1080/19490976.2024.2390680 (PMC11382725; doi:10.1080/19490976.2024.2390680)
Supplement: Supplemental Material [file KGMI_A_2390680_SM1655.zip › supplementary_Files__20_/supplementary_file_2 (1).pdf]

Supplementary file 2: Mucus mass spectrometry summary data (relative to Figure 2B)

Majority HUMAN protein IDs	FASTA headers	Score	$\log_{10}$ LFQ GAT23	$\log_{10}$ LFQ GAT27	$\log_{10}$ LFQ GAT29	$\log_{10}$ LFQ GAT23 + TNFα	$\log_{10}$ LFQ GAT27 + TNFα	$\log_{10}$ LFQ GAT29 + TNFα	$\log_{10}$ LFQ GAT23 + IL1β	$\log_{10}$ LFQ GAT27 + IL1β	$\log_{10}$ LFQ GAT29 + IL1β	$\log_{10}$ LFQ GAT23 + IFNγ	$\log_{10}$ LFQ GAT27 + IFNγ	$\log_{10}$ LFQ GAT29 + IFNγ	$\log_{10}$ LFQ GAT23 + TNFα + IL1β + IFNγ	$\log_{10}$ LFQ GAT27 + TNFα + IL1β + IFNγ	$\log_{10}$ LFQ GAT29 + TNFα + IL1β + IFNγ
**CXCL9**	C-X-C motif chemokine 9 OS=Homo sapiens OX=9606 GN=CXCL9 PE=1 SV=1	323.31	0.00	0.00	0.00	0.00	0.00	0.00	0.00	0.00	0.00	8.44	8.33	8.35	9.55	9.27	9.63
**TNF6B**	Tumor necrosis factor receptor superfamily member 6B OS=Homo sapiens OX=9606 GN=TNFRSF6B PE=1 SV=1	157.45	0.00	0.00	0.00	0.00	8.43	0.00	0.00	8.62	0.00	0.00	0.00	0.00	9.15	9.14	9.24
**X3CL1**	Fractalkine OS=Homo sapiens OX=9606 GN=CX3CL1 PE=1 SV=1	102.97	0.00	0.00	0.00	0.00	7.87	0.00	0.00	0.00	0.00	7.60	8.16	7.48	8.63	8.97	8.90
**SODM**	Superoxide dismutase [Mn], mitochondrial OS=Homo sapiens OX=9606 GN=SOD2 PE=1 SV=3	293.55	0.00	0.00	0.00	7.88	0.00	8.12	8.20	0.00	8.86	8.34	0.00	8.22	9.01	8.44	8.50

**2B11; 2B1G; DRB5**	HLA class II histocompatibility antigen, DRB1-1 beta chain OS=Homo sapiens OX=9606 GN=HLA-DRB1 PE=1 SV=2;HLA class II histocompatibility antigen, DRB1-16 beta chain OS=Homo sapiens OX=9606 GN=HLA-DRB1 PE=1 SV=1;HLA class II histocompatibility antigen, DR b	128.89	0.00	0.00	0.00	0.00	0.00	8.02	7.89	0.00	8.18	8.37	8.23	8.50	8.56	8.33	8.59
**DUOX2**	Dual oxidase 2 OS=Homo sapiens OX=9606 GN=DUOX2 PE=1 SV=2	123.28	0.00	0.00	0.00	0.00	0.00	0.00	8.25	8.21	8.41	0.00	0.00	0.00	8.62	8.03	8.54
**CATS**	Cathepsin S OS=Homo sapiens OX=9606 GN=CTSS PE=1 SV=3	107.89	0.00	0.00	0.00	0.00	0.00	7.99	8.12	0.00	8.44	8.20	0.00	8.15	8.27	0.00	8.18
**LAMB1**	Laminin subunit beta-1 OS=Homo sapiens OX=9606 GN=LAMB1 PE=1 SV=2	100.64	0.00	0.00	0.00	0.00	8.98	8.28	7.94	8.36	7.78	7.15	7.06	7.56	7.55	7.34	7.90

**C3**	Complement C3 OS=Homo sapiens OX=9606 GN=C3 PE=1 SV=2	323.31	6.98	7.15	6.78	8.55	9.98	8.92	8.55	10.07	8.35	7.23	8.90	7.45	10.42	10.32	10.17
**SYWC**	Tryptophan--tRNA ligase, cytoplasmic OS=Homo sapiens OX=9606 GN=WARS PE=1 SV=2	323.31	6.51	0.00	7.31	6.62	0.00	6.89	0.00	0.00	6.94	9.73	9.20	9.83	10.01	9.60	9.61
**ICAM1**	Intercellular adhesion molecule 1 OS=Homo sapiens OX=9606 GN=ICAM1 PE=1 SV=2	323.31	7.49	8.10	6.97	9.89	10.43	10.20	7.41	10.03	7.63	9.60	10.01	9.29	10.53	10.35	10.55
**C1R**	Complement C1r subcomponent OS=Homo sapiens OX=9606 GN=C1R PE=1 SV=2	323.31	7.67	7.53	6.97	9.08	9.69	9.37	0.00	9.71	0.00	9.27	9.76	8.97	10.16	10.10	9.97
**CXCL5**	C-X-C motif chemokine 5 OS=Homo sapiens OX=9606 GN=CXCL5 PE=1 SV=1	241.18	0.00	8.24	6.99	0.00	10.46	8.17	7.37	10.44	7.20	0.00	8.20	0.00	10.05	10.47	9.96
**CXCL3**	C-X-C motif chemokine 3 OS=Homo sapiens OX=9606	173.51	0.00	7.76	0.00	8.43	9.97	9.01	0.00	9.91	0.00	8.01	8.35	7.83	9.52	9.86	9.57

	GN=CXCL3 PE=1 SV=1																
**C1S**	Complement C1s subcomponent OS=Homo sapiens OX=9606 GN=C1S PE=1 SV=1	323.31	7.79	0.00	0.00	7.89	9.24	8.62	7.42	8.95	0.00	7.46	8.60	0.00	9.39	9.80	9.12
**IC1**	Plasma protease C1 inhibitor OS=Homo sapiens OX=9606 GN=SERPING1 PE=1 SV=2	323.31	7.72	0.00	0.00	8.05	0.00	7.76	7.77	0.00	8.67	9.99	9.27	9.74	9.73	7.98	9.37
**TIG1**	Retinoic acid receptor responder protein 1 OS=Homo sapiens OX=9606 GN=RARRES1 PE=1 SV=2	127.04	0.00	8.00	0.00	7.72	9.40	7.72	0.00	9.29	7.86	0.00	8.91	7.30	7.93	9.85	7.84
**GROA**	Growth-regulated alpha protein OS=Homo sapiens OX=9606 GN=CXCL1 PE=1 SV=1	323.31	7.68	8.69	0.00	8.85	10.33	9.38	0.00	10.32	0.00	7.72	8.02	7.71	9.65	10.34	9.82
**C4A**	Complement C4-A OS=Homo sapiens OX=9606 GN=C4A PE=1 SV=2	323.31	7.35	7.15	6.97	7.41	7.50	6.79	7.08	7.33	6.86	9.71	9.92	9.51	9.23	8.11	8.90

**GILT**	Gamma-interferon-inducible lysosomal thiol reductase OS=Homo sapiens OX=9606 GN=IFI30 PE=1 SV=3	153.65	7.07	0.00	7.08	7.80	0.00	8.30	7.53	7.39	7.87	8.98	7.75	8.70	8.82	7.74	8.58
**CERU**	Ceruloplasmin OS=Homo sapiens OX=9606 GN=CP PE=1 SV=1	323.31	7.79	8.63	7.11	9.00	9.52	9.35	9.84	10.09	10.08	8.85	9.32	8.71	9.91	9.45	9.78
**KAD2**	Adenylate kinase 2, mitochondrial OS=Homo sapiens OX=9606 GN=AK2 PE=1 SV=2	134.51	7.37	7.68	7.35	0.00	7.91	7.06	0.00	7.74	0.00	8.40	7.99	8.54	8.79	8.89	8.82
**FINC**	Fibronectin OS=Homo sapiens OX=9606 GN=FN1 PE=1 SV=4	323.31	8.06	0.00	0.00	8.86	9.09	8.87	0.00	8.90	0.00	9.06	9.33	8.93	9.01	8.61	8.94
**MUC4**	Mucin-4 OS=Homo sapiens OX=9606 GN=MUC4 PE=1 SV=4	101.87	0.00	8.04	0.00	0.00	8.05	7.90	7.93	8.66	8.00	8.41	8.43	8.02	8.53	9.03	8.37

**H14; H13**	Histone H1.4 OS=Homo sapiens OX=9606 GN=HIST1H1E PE=1 SV=2;Histone H1.3 OS=Homo sapiens OX=9606 GN=HIST1H1D PE=1 SV=2	120.14	8.00	8.24	8.22	0.00	8.20	0.00	0.00	8.32	0.00	8.43	8.72	9.06	9.08	9.53	9.30
**DLDH**	Dihydrolipoyl dehydrogenase, mitochondrial OS=Homo sapiens OX=9606 GN=DLD PE=1 SV=2	155.08	7.54	0.00	7.96	7.63	7.50	8.23	8.43	7.63	8.60	8.28	7.83	8.79	8.96	8.46	8.78
**TPM3**	Tropomyosin alpha-3 chain OS=Homo sapiens OX=9606 GN=TPM3 PE=1 SV=2	108.64	7.94	0.00	7.96	7.71	0.00	7.92	0.00	7.93	0.00	8.59	8.54	8.83	9.01	8.67	9.01

**1A03; 1A36; 1A01**	HLA class I histocompatibility antigen, A-3 alpha chain OS=Homo sapiens OX=9606 GN=HLA-A PE=1 SV=2;HLA class I histocompatibility antigen, A-36 alpha chain OS=Homo sapiens OX=9606 GN=HLA-A PE=1 SV=1;HLA class I histocompatibility antigen, A-1 alpha chain O	258.07	7.94	0.00	8.26	8.11	0.00	7.70	7.34	0.00	0.00	8.87	7.45	9.12	9.18	8.89	9.16
**TPM4**	Tropomyosin alpha-4 chain OS=Homo sapiens OX=9606 GN=TPM4 PE=1 SV=3	323.31	8.24	8.31	8.40	7.59	8.58	8.36	7.54	8.54	7.50	9.06	8.94	9.27	9.44	9.36	9.54
**FLNB**	Filamin-B OS=Homo sapiens OX=9606 GN=FLNB PE=1 SV=2	323.31	8.20	0.00	8.49	8.14	7.59	8.53	7.55	7.52	7.88	9.32	8.64	9.33	9.47	9.02	9.36
**VINC**	Vinculin OS=Homo sapiens OX=9606 GN=VCL PE=1 SV=4	169.60	8.05	0.00	0.00	0.00	0.00	8.08	7.65	8.26	0.00	8.67	8.34	8.69	8.69	8.56	8.79

**LAMA3**	Laminin subunit alpha-3 OS=Homo sapiens OX=9606 GN=LAMA3 PE=1 SV=2	188.00	7.49	0.00	7.89	7.50	8.02	8.09	7.94	8.36	7.96	8.09	7.63	7.99	8.57	8.33	8.89
**OLFM4**	Olfactomedin-4 OS=Homo sapiens OX=9606 GN=OLFM4 PE=1 SV=1	323.31	8.66	8.50	8.01	10.53	9.41	10.06	9.85	9.84	10.22	7.74	7.96	7.83	9.77	9.33	9.46
**CFAH**	Complement factor H OS=Homo sapiens OX=9606 GN=CFH PE=1 SV=4	323.31	6.29	8.83	0.00	7.21	9.54	6.87	0.00	9.57	0.00	9.22	10.32	9.10	9.37	9.67	9.03
**LAMB3**	Laminin subunit beta-3 OS=Homo sapiens OX=9606 GN=LAMB3 PE=1 SV=1	230.67	7.52	0.00	7.95	7.61	8.08	8.39	8.10	8.23	7.92	8.19	7.45	8.13	8.62	8.10	8.94
**ROA2**	Heterogeneous nuclear ribonucleoproteins A2/B1 OS=Homo sapiens OX=9606 GN=HNRNPA2B1 PE=1 SV=2	122.62	8.06	7.10	7.88	6.90	7.88	8.18	7.16	7.95	7.18	8.49	7.91	8.74	8.44	9.04	8.94
**LTF**	Lactotransferrin OS=Homo sapiens OX=9606 GN=LTF PE=1 SV=6	323.31	9.92	10.75	9.66	11.78	12.20	11.82	12.04	12.19	11.95	9.59	10.52	9.60	11.33	11.61	11.11

**SDCB2**	Syntenin-2 OS=Homo sapiens OX=9606 GN=SDCBP2 PE=1 SV=2	135.67	7.50	0.00	0.00	8.34	7.32	8.37	8.78	7.96	8.90	7.80	0.00	7.34	8.30	0.00	8.15
**SODC**	Superoxide dismutase [Cu-Zn] OS=Homo sapiens OX=9606 GN=SOD1 PE=1 SV=2	323.31	8.47	8.67	7.66	8.51	8.99	8.77	9.07	8.81	9.11	8.99	8.89	9.12	9.41	9.47	9.48
**SPTN1**	Spectrin alpha chain, non-erythrocytic 1 OS=Homo sapiens OX=9606 GN=SPTAN1 PE=1 SV=3	131.91	0.00	0.00	7.78	0.00	0.00	7.53	0.00	7.46	0.00	8.26	7.81	8.56	8.31	7.88	8.49
**C2**	Complement C2 OS=Homo sapiens OX=9606 GN=C2 PE=1 SV=2	306.50	0.00	8.58	0.00	0.00	8.55	7.64	0.00	8.63	0.00	9.09	9.52	8.74	9.03	8.83	8.81
**LAMC1**	Laminin subunit gamma-1 OS=Homo sapiens OX=9606 GN=LAMC1 PE=1 SV=3	140.17	7.37	0.00	7.49	8.17	8.75	8.34	8.37	8.68	8.18	7.49	7.20	7.26	7.78	7.96	7.86
**LCN2**	Lipocalin 2. Neutrophil gelatinase-associated lipocalin OS=Homo sapiens OX=9606	323.31	10.86	10.33	10.39	11.55	11.02	11.41	11.82	11.26	11.82	10.91	10.57	10.76	11.13	10.43	10.93

	GN=LCN2 PE=1 SV=2																
**FCGBP**	IgGFc-binding protein OS=Homo sapiens OX=9606 GN=FCGBP PE=1 SV=3	323.31	10.14	10.14	9.83	10.62	10.63	10.66	11.18	10.92	11.40	9.91	10.19	9.78	9.96	9.94	9.77
**LAMA5**	Laminin subunit alpha-5 OS=Homo sapiens OX=9606 GN=LAMA5 PE=1 SV=8	149.53	7.48	0.00	7.64	8.35	8.70	8.89	8.25	8.41	8.39	7.67	0.00	0.00	7.51	0.00	7.20
**ZG16B**	Zymogen granule protein 16 homolog B OS=Homo sapiens OX=9606 GN=ZG16B PE=1 SV=3	146.65	8.15	0.00	7.36	9.34	0.00	8.35	8.30	6.97	9.90	7.87	0.00	6.86	7.58	0.00	0.00
